# Asthma and Asthma Medication Are Common among Recreational Athletes Participating in Endurance Sport Competitions

**DOI:** 10.1155/2018/3238546

**Published:** 2018-06-21

**Authors:** Amanda Näsman, Tommie Irewall, Ulf Hållmarker, Anne Lindberg, Nikolai Stenfors

**Affiliations:** ^1^Department of Public Health and Clinical Medicine, Unit of Medicine–Östersund, Umeå University, 90187 Umeå, Sweden; ^2^Department of Internal Medicine, Mora Hospital, 79285 Mora, Sweden; ^3^Department of Public Health and Clinical Medicine, Unit of Medicine–Sunderbyn, Umeå University, 90187 Umeå, Sweden

## Abstract

**Background:**

Asthma prevalence is high among elite endurance athletes, but little is known about its prevalence among competitive recreational athletes. The aim of this study was to determine the prevalence of self-reported asthma and asthma medication use among competitive recreational endurance athletes and their association with training.

**Methods:**

A web survey on asthma and medication was conducted among 38,603 adult participants of three Swedish endurance competitions (cross-country running, cross-country skiing, and swimming).

**Results:**

The overall response rate was 29%. The prevalence of self-reported asthma (physician-diagnosed asthma and use of asthma medication in the last 12 months) was 12%. Among those reporting asthma, 23% used inhaled corticosteroids and long-acting beta-agonists daily. We found no association between training volume and daily use of asthma medication, except a trend in relation to short-acting beta-agonists. Independent predictors of self-reported asthma were female sex, allergic rhinitis, previous eczema, family history of asthma, cycling, and training for >5 h 50 min/week.

**Conclusions:**

The prevalence of self-reported asthma among Swedish competitive recreational endurance athletes appears to be higher than that in the general Swedish population. A large proportion of recreational athletes were reported with asthma use medications, indicating an association between high physical activity and self-reported asthma among competitive recreational athletes.

## 1. Introduction

Asthma is a chronic inflammatory airway disease characterized by repeated episodes of bronchial constriction with wheezing, tightness of the chest, coughing, and breathlessness. The prevalence of asthma among adults in Sweden is estimated at 9–11% [1, 2]. A previous study has shown vigorous physical activity to be independently associated with an increased risk of asthma in a general population sample [3]. Moreover, the prevalence of asthma, or airway hyperresponsiveness, was found to be higher among elite athletes than among the general population [4]. The main hypothesis to explain these findings is that asthma among endurance athletes is associated with high-volume ventilation of dry air [4]. In keeping with this idea, asthma prevalence is high among athletes in endurance sports that require intense breathing, such as long-distance running and skiing [5, 6]. The season of competition does not seem to influence asthma prevalence among elite athletes [7]. The prevalence of asthma among elite competitive swimmers is also high [8], and high-volume ventilation and inhalation of chlorine derivatives are believed to be underlying factors [9]. Other predictors of asthma among athletes are female sex, atopy, and a family history of asthma [6, 10]. In fact, a recent study has found evidence for two distinct phenotypes of asthma in elite athletes: *atopic asthma*, defined by allergy and rhinitis, and *sports asthma*, defined by respiratory symptoms and airway hyperresponsiveness without allergic features [11].

Studies of asthma prevalence in athletes have predominantly been conducted among elite athletes [4], whereas those conducted with competitive recreational athletes have been few and mostly rather small. In a study including 484 US runners, 17% reported asthma [12], while among 95 recreational French endurance athletes, 4% reported physician-diagnosed asthma [13]. In a Norwegian study from 2001, the prevalence of asthma among nonelite runners (*n*=512) and skiers (*n*=315) was 7% and 11%, respectively [14].

The aim of this cross-sectional study was to determine the prevalence of self-reported physician-diagnosed asthma and asthma medication use among competitive recreational endurance athletes and their association with training. These athletes were participating in three large Swedish endurance competitions open to the public, which included three different endurance sports: cross-country running (CCR) Lidingöloppet, cross-country skiing (CCS) Vasaloppet, and open-water swimming (OWS) Vansbrosimningen.

## 2. Materials and Methods

### 2.1. Study Design

The study was conducted as an internet-based web survey.

### 2.2. Participants and Setting

**Figure 1:**
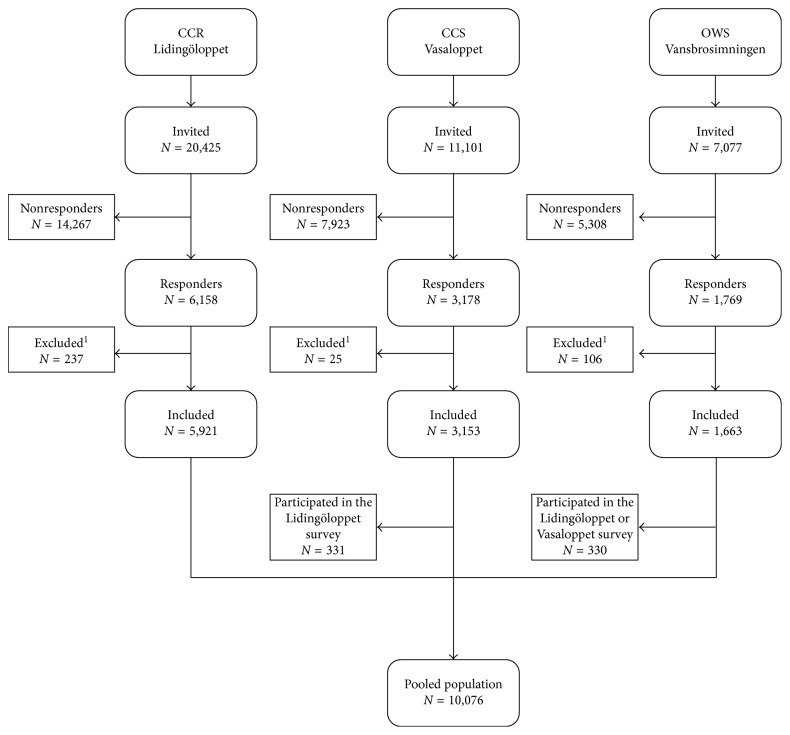
Flow chart of study participants from the three large Swedish public endurance competitions: cross-country running (CCR Lidingöloppet), cross-country skiing (CCS Vasaloppet), and open-water swimming (OWS Vansbrosimningen). ^1^Age < 18 years/unknown age.

Swedish citizens of 18 years or older registered for participation in any of the three public endurance competitions (CCR Lidingöloppet in 2014; CCS Vasaloppet and OWS Vansbrosimningen in 2015) and with an e-mail address were eligible for inclusion (Table 1). CCR Lidingöloppet is a 30 km cross-country running competition in Stockholm and was held on September 28 in 2014. A total of 20,425 entrants in this competition were invited to participate in the study. The 90 km CCS Vasaloppet is described as the world's oldest, largest, and longest cross-country skiing competition, held on the second Sunday in March in the county of Dalarna; in 2015, it was held on March 8. A total of 11,101 entrants in this competition were invited to complete the survey. At 3 km, OWS Vansbrosimningen is the largest open-water swimming competition in Sweden and was held on July 4 in 2015. A total of 7077 participants in this competition were invited to complete the survey. All three competitions are open to recreational and elite professional athletes from all countries. A flow chart of the recruitment of the study population is given in Figure 1. All participants signed written informed consent forms, and the study was approved by the Regional Ethical Review Board in Umeå.

### 2.3. Methods

The study population received an invitation to the online survey (Textalk AB, Sweden; https://websurvey.textalk.se/se) by e-mail one month before the competition, and a reminder was sent to the nonresponders after 2 weeks. The questionnaire was based on ECRHS II [15], a validated international questionnaire including questions about airway symptoms, allergic rhinitis, and asthma, and questions regarding training, sport, and asthma medication additionally were included.

Study variables of special interest were queried, as follows:

  “Physician-diagnosed asthma”: “Have you ever had asthma?” and “Was the diagnosis made by a physician?”
  “Self-reported asthma”: physician-diagnosed asthma and “Have you used any asthma medication, including inhalers, sprays, or pills in the last 12 months?”
  “Training” was defined by the question, “In the last 12 months, how much time did you spend during an ordinary week on exercise that made you out of breath; for example, running, aerobics, or ball sport?” Training volume is presented as hours and minutes per week.
  “Sport” was defined by the question, “During the last 12 months, on which type of exercise did you spend the most hours of training?” with the alternatives “running,” “cycling,” “skiing,” “swimming,” and “others.”
  “Family history of asthma”: “Have any of your parents or siblings had asthma?”
  “Allergic rhinitis”: “Do you have hay fever or other allergies with symptoms from the eye and/or nose?”
  “Current smoker” was defined as answering “yes” to the questions, “Have you ever smoked one or more cigarettes per day during at least one year?” and “Have you smoked during the last month?”
  “History of eczema”: “Have you ever had eczema or any kind of skin allergy?”

Asthma medication use in the last 12 months was categorized into short-acting or long-acting beta-agonists (SABA/LABA), inhaled corticosteroids (ICS), and ICS + LABA fixed combination (ICS + LABA). To aid participants in categorizing their medicine, several trade names were used as examples. Medication usage for the last 12 months was ordinally categorized into “never,” “sometimes,” “less than 2 months,” “more than 2 months,” and “daily.”

*Self-reported asthma* was furthermore categorized into the following:

 
*Intermittent*: use of SABA/LABA sometimes or less/more than 2 months *and* no use of ICS or ICS/LABA
 
*Uncontrolled intermittent*: daily use of SABA/LABA *and* no use of ICS or ICS/LABA
 
*Persistent*: daily use of ICS or ICS/LABA
 
*Uncontrolled persistent*: daily use of SABA/LABA *and* daily use of ICS or ICS/LABA.

### 2.4. Statistics

Results were analyzed for each competition separately and for all participants pooled together. Athletes in the latter two surveys (from CCS Vasaloppet and OWS Vansbrosimningen) stating that they had participated in the survey previously were excluded from the pooled population, so analyses ultimately included 10,076 unique athletes. Student's *t*-test and the Pearson chi-square test were used for group comparisons of continuous and categorical variables, respectively. The variable representing training (hours/week) was skewed. The study population was therefore transformed into quartiles (<3 h, 3 to <4 h, 4–5 h 50 min, >5 h 50 min) to represent low, low-intermediate, intermediate-high, and high training volumes. The Pearson chi-square test was used to compare the amount of training (as a categorical variable) between athletes with self-reported asthma with and without daily asthma medication in the last 12 months. In order to identify professional athletes at the international or high national level, we chose to define elite/semielite athletes by an arbitrary cutoff of ≥10 training hours/week.

Adjusted odds ratios for the presence of self-reported asthma and their 95% confidence intervals for the pooled study population were estimated using a binary logistic regression model. Age dichotomized at the median (42 years), sex, amount of training/week in quartiles, sport, allergic rhinitis, eczema, and family history of asthma were included as risk factors. Skewed data were presented using medians. A *p* value < 0.05 was considered statistically significant.

## 3. Results

The response rates were 30% (5,921/20,425) for CCR Lidingöloppet, 29% (3,153/11,101) for CCS Vasaloppet, and 25% (1,663/7,077) for OWS Vansbrosimningen, giving an overall response rate of 29%.

### 3.1. Asthma Prevalence in the Three Competitions and Differences between Sexes

A summary of the characteristics of the participants in each of the competitions is presented in Table 2. The prevalence of physician-diagnosed asthma ranged from 17 to 20%, and between 11% and 14% had self-reported asthma. Women were in the minority in all three competitions, representing 29% of the pooled study population. A higher proportion of women than men had physician-diagnosed asthma, self-reported asthma, and a family history of asthma (*p* < 0.001) in CCR Lidingöloppet and CCS Vasaloppet, but not in OWS Vansbrosimningen. The proportion of athletes training ≥10 h per week was 3% (*n*=201) in CCR Lidingöloppet, 7% (*n*=219) in CCS Vasaloppet, and 9% (*n*=104) in OWS Vansbrosimningen.

### 3.2. Pooled Study Population

In the pooled population, 3% reported swimming, 19% cycling, 57% running, 14% skiing, and 7% other sports as their main physical activity. In total, 12% had self-reported asthma, which had a prevalence of 14% among cyclists, 12% among skiers, 11% each among runners and swimmers, and 13% among athletes with main training in other sports. The overall proportion of athletes training ≥10 h per week was 5% (*n*=470). Female athletes (29%) were younger than the males (39 versus 42 years) and reported more physician-diagnosed asthma (21% versus 16%), more self-reported asthma (15% versus 10%), family history of asthma (25% versus 20%), and shortness of breath following strenuous exercise (13% versus 5%) compared to the males (all *p* < 0.001).

### 3.3. Use of Asthma Medication among Swedish Competitive Recreational Athletes

In the pooled population, the proportion of athletes reporting daily usage of SABA/LABA, ICS, or ICS + LABA was 3% for each medication category. Among athletes with self-reported asthma, 23% underwent daily maintenance therapy with ICS + LABA. Only 7% reported daily use of a leukotriene receptor antagonist, and further data of this are not presented. A total of 21% of the runners in CCR Lidingöloppet, 28% of the skiers in CCS Vasaloppet, and 24% of the swimmers in OWS Vansbrosimningen used ICS + LABA daily (Table 3). In the group that trained the least (<3 h/week), 18% reported daily usage of SABA/LABA compared to 27% among those who trained the most (>5 h 50 min; *p*=0.05). There was no significant difference in daily use of ICS and ICS + LABA between the groups that trained the least and the most.

Among athletes with self-reported asthma, 212 (18%) had *intermittent asthma* and 36 (3%) fulfilled the study criteria of *uncontrolled intermittent asthma*. A total of 514 athletes (46%) had *persistent asthma* and 171 (15%) fulfilled the study criteria for *uncontrolled persistent asthma*. The proportion of athletes in each category did neither differ between the groups that trained the least and the most nor between main sports.

### 3.4. Risk Factors Associated with Self-Reported Asthma

Of athletes with self-reported asthma, 71% had allergic rhinitis. No significant differences in the prevalence were found between sports. The following variables were individually associated with self-reported asthma: female sex, age <42 years, allergic rhinitis, history of eczema, family history of asthma, training more than 5 h 50 min per week, and having cycling as the main sport. In the multivariate model, all these variables remained as independent risk factors for self-reported asthma, except age <42 years (Table 4).

## 4. Discussion

In this large internet-based survey including 10,737 (10,076 unique) athletes participating in the three major Swedish endurance competitions, the overall prevalence of self-reported asthma was 12%. Among the athletes with asthma, 22% used SABA/LABA, 25% used ICS, and 23% used ICS + LABA daily. Female sex, allergic rhinitis, previous eczema, family history of asthma, cycling as the main sport, and training more than 5 h and 50 min per week were independent predictors of self-reported asthma. There was no statistically significant association between training volume and daily use of asthma medication, except a trend in relation to short-acting beta-agonists. To the best of our knowledge, this is the largest survey on asthma among athletes ever conducted.

Comparisons between the current results and previous studies are complicated by the difficulty in defining the competitive recreational athlete. We were unable to find in the literature a standardized level of training volume to discriminate between elite and recreational endurance athletes. However, based on our experience, Swedish cross-country skiers at the international or highest national level train at least 10 h or more per week on a yearly basis. In the current study population, only 5% reported a training volume of at least 10 h per week during the last 12 months and could thus be considered elite rather than recreational endurance athletes.

In the present study, the prevalence of self-reported asthma was slightly higher than that in previous studies among amateur athletes, which have a reported prevalence between 4% and 10% [13, 14]. On the contrary, in 2006, the prevalence of asthma among 484 US recreational road runners was estimated at 17% [12].

The criteria for therapeutic use exemptions regarding asthma medication have shifted to a somewhat more tolerant attitude about the use of beta-2-agonists. Among 150 US recreational road runners reporting asthma symptoms, only 9 (6%) had been prescribed asthma medication [12]. Among male endurance athletes in the south of France, 1% had used asthma medication [13].

On the one hand, the proportion of athletes with self-reported asthma in this study was slightly lower than that previously reported among Nordic elite athletes. A total of 14% of Finnish Olympic and elite track and field athletes [5, 16] and 25–28% of Swedish elite cross-country skiers reported use of asthma medication [6]. On the other hand, it has been suggested that there is undertreatment of asthma among Danish national team athletes because only 7% report current use of asthma medication [17]. However, the use of asthma medication among Swedish competitive endurance athletes appears to be slightly higher than that in the Swedish general population (9–11%) [1, 18, 19].

Asthma maintenance medication is based on daily use of ICS. In the present study, 3% of the athletes reported daily use of fixed-combination ICS + LABA. Among those with self-reported asthma, daily use of ICS + LABA was at 23%, slightly higher than the general population's rate of 19% [19]. Whether this increased use of ICS + LABA arises from more severe disease, overuse, or higher physical demands among athletes remains unknown. The recreational athletes in the present study had a slightly lower use of asthma medication compared to elite athletes, such as Canadian athletes with asthma, of whom 34% used ICS + LABA [20]. In 2009, 4% of Finnish Olympic athletes used a fixed combination of ICS + LABA [16]. Among Danish elite athletes using asthma medication, 67% inhaled beta-2-agonists + ICS [17]. Taken together, these findings suggest that vigorous physical training at the elite and competitive recreational levels does not exclude athletes with moderate-to-severe asthma needing daily ICS + LABA. On the contrary, asthmatic subjects with regular high physical activity may need a higher level of asthma medication, that is, ICS + LABA, to pursue their main sport of interest.

We observed a trend towards increased use of SABA/LABA among athletes training >5 h 50 min per week (*p*=0.05). One would expect an increased use of SABA, as prevention against exercise-induced asthma, among athletes training regularly. On the contrary, an increased use of SABA/LABA is an indication, for the treating physician, to introduce or increase the use of controller medication, such as ICS, and thus reduced the need for SABA. Elite cross-country skiers with asthma do not appear to use more SABA/LABA than a reference group of patients with asthma [21].

Female sex, allergic rhinitis, eczema, and family history are known predictors of asthma in the general population [1, 3, 18] and among elite athletes [6, 10, 17]. Allergic rhinitis is very common among athletes [22]. Besides these common risk factors, including allergic rhinitis, we found also cycling and training >5 h 50 min per week as independent predictors of self-reported asthma (Table 4). Training > 20 h per week is an independent predictor of asthma among elite athletes [10], and in a general population sample, training ≥ 2 times and ≥7 h per week has been associated with an increased risk of asthma [3].

A distinct phenotype of asthma has been identified in elite athletes: *sports asthma*, defined by exercise-induced respiratory symptoms and airway hyperresponsiveness without allergic features. Water and winter sport athletes have an increased risk of “sports asthma” [11]. Our clinical experience is that many cases with asthma among Swedish elite cross-country skiers do not “behave” like cases with ordinary allergic asthma. Unfortunately, as we in the present study lack data on eNO, airway hyperresponsiveness/reversibility, and atopy, we cannot use the present results to neither confirm nor dispute the results by Couto et al. [11]. In the present results, allergic rhinitis was equally prevalent, ca. 70%, in swimmers and skiers.

Among the athletes at the international or high national competitive level with a high training volume are probably many professional or semiprofessional athletes. In contrast to training volume, age was not independently associated with risk of asthma. High training volume thus appears to be a stronger predictor of self-reported asthma than age, suggesting that training might affect remission of asthma among adult athletes. Cycling as the main sport was an independent predictor of self-reported asthma, and a high asthma prevalence has previously been reported among Olympic cyclists [4]. Cycling requires long training sessions with high-volume breathing in an outdoor environment, often along roads with exposure to vehicle exhaust, tire particles, and pollen. These environmental factors may contribute to the observed association between asthma and cycling as a main sport.

The strengths of our study are the very large study sample, the inclusion of three different endurance sports, and the use of a validated questionnaire. However, the rather low response rate of 29% carries the risk of selection bias. We are not aware of any other web surveys on airway symptoms or asthma among athletes. A postal questionnaire instead of a web survey or a postal reminder as a follow-up might have increased the response rate, but we had access only to e-mail and not postal addresses. In the previously cited US road runner study, only 11% of athletes who were surveyed on the race day participated [12], while the response rate was 68% in the Norwegian study in which the postal questionnaire was sent to participants 2 months after the races [14]. In the current investigation, athletes answered the questionnaire prior to competition to avoid the influence of airway symptoms occurring during the races. On the contrary, surveying athletes before racing could have resulted in including athletes who did not complete the race. The selection bias is difficult to assess, but in a Swedish questionnaire study with a 62% response rate, there was no difference in respiratory symptoms and asthma when comparing responders and nonresponders [23]. An Italian study, however, found that subjects with self-reported asthma and asthma-like symptoms may be more prone to responding to the ECRHS questionnaire [24].

Some of the athletes with self-reported asthma may have been misdiagnosed and suffered from other respiratory diseases, such as exercise-induced laryngeal obstruction [25] or dysfunctional breathing. Some might even have had chronic obstructive pulmonary disease (COPD), but we consider the probability to be low that people with COPD would participate in these types of competitions. The study was cross-sectional, giving us the opportunity to assess associations but not cause and effects.

## 5. Conclusions

The prevalence of self-reported asthma among Swedish competitive recreational endurance athletes appears to be higher than that in the general Swedish population. A large proportion of recreational athletes with asthma use ICS + LABA. The results support an association between high physical activity and self-reported asthma among competitive recreational athletes. Training volume should therefore be thoroughly surveyed in recreational athletes with exercise-induced breathlessness. Further research on how to prevent asthma among endurance athletes is justified.

## Figures and Tables

**Table 1:** Description of the participants in the three Swedish large endurance competitions: CCR Lidingöloppet in 2014 and CCS Vasaloppet and OWS Vansbrosimningen in 2015.

Event	CCR Lidingöloppet	CCS Vasaloppet	OWS Vansbrosimningen
Registered participants (*n*)	21,507^2^	15,800^3^	8,605^4^
Women (%)	27	14	35
Completed race (*n*)	16,363	12,625	7,845
Age (median, IQR)	40 (32–47)	41 (32–49)	NA
Race time (median, range)^1^	3:00 (1:42–6:12)	8:36 (4:07–12:49)	NA

^1^Units: hours:minutes. ^2,3,4^Of these registered participants, a total of 20,425^2^, 11,101^3^, and 7,077^4^ were Swedish citizens, of age 18 or above, and had registered an e-mail address. IQR: interquartile range; NA: not applicable.

**Table 2:** Basic characteristics of the study population in each of the three competitions: CCR Lidingöloppet, CCS Vasaloppet, and OWS Vansbrosimningen, and the pooled study population.

	CCR Lidingöloppet (*n*=5,921)		CCS Vasaloppet (*n*=3,153)		OWS Vansbrosimningen (*n*=1,663)		Pooled (*n*=10,076^2^)	
	*N*	*N* (%)	*N*	*N* (%)	*N*	*N* (%)	*N*	*N* (%)
Age^1^	5,921	41 (34–48)^1^	3,153	42 (33–50)^1^	1,663	42 (33–50)^1^	10,076	42 (34–49)^1^
Female	5,790	1,764 (30)	3,083	577 (19)	1,598	706 (44)	9,827	2,849 (29)
Current smoker	5,893	108 (2)	3,149	31 (1)	1,661	39 (2)	10,043	167 (2)
Physician-diagnosed asthma	5,824	979 (17)	3,138	522 (17)	1,651	310 (20)	9,954	1,692 (17)
Self-reported asthma^3^	5,823	658 (12)	3,136	341 (11)	1,650	214 (14)	9,950	1,135 (12)
Allergic rhinitis	5,921	2,161 (37)	3,153	1,024 (33)	1,661	559 (35)	10,074	2,503 (36)
Family history of asthma	5,531	1,185 (22)	2,979	608 (21)	1,562	351 (23)	9,456	1,987 (22)
Training^1^ (hours:minutes/week)	5,848	4:00 (2:40–5:00)^1^	3,012	5:00 (3:00–6:00)^1^	1,125	4:00 (3:00–6:00)^1^	9,419	4:00 (3:00–5:30)^1^

^1^Age and training presented as medians with interquartile ranges. ^2^Only unique subjects included in the pooled study population. ^3^Physician-diagnosed asthma and use of asthma medication during the last 12 months. *N* denotes the number of answers.

**Table 3:** Self-reported frequency of asthma medication use among Swedish competitive recreational athletes with self-reported asthma, that is, physician diagnosis of asthma and use of asthma medication during the last 12 months, in each of the competitions and pooled data.

		Never	Sometimes	<2 months	>2 months	Daily
CCR Lidingöloppet	*SABA/LABA*	98 (15)	295 (44)	40 (6)	83 (12)	154 (23)
	*ICS*	319 (48)	96 (15)	41 (6)	40 (6)	166 (25)
	*ICS* + *LABA*	439 (67)	34 (5)	16 (2)	26 (4)	139 (21)

CCS Vasaloppet	*SABA*/*LABA*	53 (16)	178 (52)	15 (4)	31 (9)	64 (19)
	*ICS*	182 (55)	53 (16)	18 (5)	19 (6)	62 (18)
	*ICS* + *LABA*	203 (60)	22 (7)	9 (3)	9 (3)	93 (28)

OWS Vansbrosimningen	*SABA/LABA*	22 (10)	99 (45)	15 (7)	21 (10)	63 (29)
	*ICS*	97 (46)	19 (9)	8 (4)	8 (4)	79 (37)
	*ICS* + *LABA*	148 (69)	7 (3)	3 (1)	3 (1)	52 (24)

Pooled study population	*SABA*/*LABA*	167 (15)	531 (46)	67 (6)	126 (11)	259 (22)
	*ICS*	563 (50)	153 (14)	64 (6)	63 (5)	283 (25)
	*ICS* + *LABA*	736 (66)	61 (6)	27 (3)	36 (3)	262 (23)

SABA = short-acting beta-2-agonists; LABA = long-acting beta-2-agonists; ICS = inhaled corticosteroids. Data are presented as *n* (%).

**Table 4:** Factors associated with self-reported asthma in the pooled study population of 10,076 Swedish competitive recreational athletes.

	Unadjusted			Adjusted		
	OR	95% CI	*p* value	OR	95% CI	*p* value
Female sex^1^	1.56	1.37–1.77	**<0.001**	1.64	1.40–1.92	**<0.001**
Age >42 years^2^	0.87	0.77–0.98	**0.022**	1.02	0.88–1.19	0.766
Allergic rhinitis	5.53	4.84–6.33	**<0.001**	5.25	4.48–6.16	**<0.001**
History of eczema	1.85	1.64–2.12	**<0.001**	1.24	1.07–1.46	**0.004**
Family history of asthma	3.39	2.97–3.87	**<0.001**	2.76	2.36–3.22	**<0.001**
*Training* ^3^						
3–4 h	1.09	0.91–1.32	0.443	1.08	0.87–1.35	0.718
4 h–5 h 50 min	1.14	0.95–1.37	0.277	1.13	0.91–1.40	0.503
>5 h 50 min	1.47	1.23–1.76	**<0.001**	1.48	1.20–1.86	**<0.001**
*Sport* ^4^						
Swimming	1.04	0.70–1.50	0.921	0.75	0.43–1.25	0.296
Cycling	1.34	1.14–1.56	**<0.001**	1.25	1.03–1.51	**0.024**
Skiing	1.13	0.93–1.35	0.158	1.16	0.92–1.45	0.204
Others	1.22	0.96–1.54	0.095	0.91	0.67–1.23	0.562

^1^Reference: “male.” ^2^Reference: “age < 42 years.” ^3^Training/week categorized into quartiles; reference quartile 1: <3 h/wk. ^4^Reference: “running.” Significant values are in bold.

## Data Availability

Data will not be made available as study participants were informed that their answers would not be shared.
